# 

**Published:** 1891-02-28

**Authors:** 


### NOTICE TO CORRESPONDENTS.

All MS., Letters, Books for Review, and other matters intended for the Editor should be addressed The Editor, The Lodge, Porchester Square, London, W.

The Editor cannot undertake to return rejected M.S., even when accompanied by stamped directed envelope.

**Notice to Advertisers and Subscribers.**

All Advertisements, Orders for Copies of The Hospital, and Business Communications should be addressed to The Publisher, *not to the Editor*, The Hospital, 140, Strand, W.C.

The Cost of Subscription, post free, is as follows:—Per week, 1½d.; per quarter, 1s. 8d.; per half-year, 3s. 3d.; per annum, 6s. 6d. Foreign:—Per annum, 8s. 8d.; payable, in all cases, in advance.

### General Charities.

[This Column is devoted to an account of work of charitable institutions other than Medical Charities. The Editor will be glad to receive reports or news of work at 140, Strand. *Philanthropy* should be plainly written on the envelope.]

**THE ROYAL SOCIETY FOR PREVENTION OF CRUELTY TO ANIMALS.**—Few facts go to show more clearly the advance during the present century of practical humanitarianism than the progress made in popular favour by this Society, which has its office at 105, Jermyn-street, S.W., The difficulties with which this institution had to contend nearly seventy years ago were such as no one of the present generation can fully appreciate. It is little to the credit of either the hearts or the understanding of our forefathers that the sight of suffering in an animal was a source of enjoyment, which in a more enlightened age is rightly held to be a mark of the lowest mental degradation. When bull-baiting, badger-baiting, cock-fighting, and other so-called sports were the pastime of the presumably refined and intellectual section of society, it was idle to expect that the condition of animals subjected to heedless brutality and wanton torture should excite the commiseration of an ignorant and thoughtless public. At the beginning of the present century the general treatment of animals was inhuman to a degree which would have caused a feeling of pious horror not unmingled with a pardonable sense of superiority in the breast of an ancient Roman. To have appealed to a votary of badger-baiting to show compassion and mercy to one of the lower animals would have been to arouse scorn, ridicule, and indignation. How literally true this is and how widespread was the prevailing indifference of men to the feelings of animals is evidenced only too clearly by the insensibility displayed in the House of Lords to the demands of Lord Erskine on behalf of suffering brutes. It was against such a mental state in all ranks of society that the pioneers and founders of the Institution had to contend, and its success is the best proof of the commonsense and discretion displayed by the pioneers of the movement in the face of constant contumely and ignorant ridicule. A Society, the chief aim and object of which is to prosecute and punish offenders, may be said to create for itself at the outset almost insuperable difficulties, and to be in danger of arousing a storm of unpopularity whose troubled waters no oil of discretion can pacify. It is much easier to excite a feeling of compassion in the public breast, in these days especially, for the vilest murderer and the most cruel torturer than to arouse a sense of injustice in the treatment of a dumb creature. The price of a martyr's crown is not extortionate, it is within the means of the humblest of her Majesty's subjects, but whether it sits with the uneasiness commonly alleged as the characteristic of the pose of the monarch's headgear we cannot vouch. Had the Institution in its earlier years employed less activity in the prosecution of offenders, it would no doubt have met with fewer difficulties to overcome. The Society is to be congratulated upon possessing a management whose zeal has been tempered with discretion, and, if in past years too much attention was paid to the curative aspect of its operations, in recent years it has devoted its energies and funds more fully to the preventive aspect. From its earliest youth a child seems prone to destruction; the capture of a barn-door fowl and the plucking of its feathers will prove more fascinating to the untutored infant than the nursing of an imaginary offspring in the shape of a doll. In fact the destructive nature of a child is too often shown in the wanton way in which it drains the life blood or rather the sawdust from the unfortunate doll's body and tears it ruthlessly limb from limb. The untaught child is innocent of the knowledge that a mouse's tail exists for the benefit of the mouse and not for the purpose of being used as a pendulum for swinging the mouse for the torturer's especial delectation. Recognizing the presence of this sad flaw in budding human nature, the Society now devotes much time and attention to the promotion of humane education, and its efforts in this direction are carried out by a committee of ladies. As an elevator of human nature society at large owes some debt of gratitude to those who first began the work, as well as to those who continue it. Thoughtlessness often rather than innate cruelty, often leads to acts which would never be committed by those whose early training has not been neglected. The doctrine that we should do to others as we would they should do to us was not meant to apply solely to our treatment of human beings; it has its application with equal force to the treatment of dumb creation, and whether at home or at school, children cannot be practised too early or too often in acts of kindness to animals, or be discouraged too frequently in amusements which inflict pain on anything that has life. Few things tend more to the refinement of the human mind than the love of nature, and those who have learnt to love nature will seldom, or ever, be found wanting in compassion for animals. Formerly cruelty was the privilege of all classes of society; it is now, in the main, confined to the costermonger and hodge, whose dulness of intellect is only too often accentuated by the fact that they have turned themselves into peripatetic beer barrels or gin distilleries. In its capacity as an educational agency, the institution disseminates in schools and amongst owners of animals its monthly journals, the *Animal World* and *Band of Mercy*, and other papers which inculcate the duty of kindness; in addition to this, subjects for essays are sent every year to the five thousand and more schools in the metropolis, to be written by the children and teachers in the hope that the occupation of the minds of the young in thinking and writing on the matter will not only promote the object of the Society, but sow the seed which shall grow into the tree of refinement. We are not aware whether the Society publishes any of these essays; probably not, but doubtless some of them would prove a valuable addition to the humorous and comical literature of the day.

### Scraps and Gleanings.

Small-pox has broken out amongst the Royal Irish Constabulary in Belfast and cases have been sent, to the hospital.

The Queen has forwarded a further contribution of twenty guineas to the East London Hospital for Children, Shadwell.

At a meeting of working men in Birmingham, the collection taken in aid of the new General Hospital amounted to £49.

The Duke of Northumberland has sent forty pheasants for the use of the patients in the Hospital for Epilepsey and Paralysis.

Dr. Marcus Fay, the Hungarian physician, states that he has completely cured a case of cancer by the application of aniline.

Sir Julian Goldsmid will preside at the festival dinner of the Metropolitan Hospital, to be held on April 21st, at the Whitehall Rooms.

Mr. G. H. Horsfall has retired from the office of president of the Royal Southern Hospital, Liverpool, which he has filled for 27 years.

A woman has died from the effect of chloroform, which was administered by a doctor with the object of making a necessary surgical examination.

The House Committee of the Cancer Hospital, Brompton, have arranged to set aside a certain number of beds for treatment under Dr. Koch's method.

A site has been selected in Perranporth for the Convalescent Home which Mr. Passmore Edwards has promised to build and present to the county of Cornwall.

Dr. Bernheim is continuing his experiments with the transfusion of blood in cases of tuberculosis, and will also apply his system to persons suffering from anæmia.

Many of the soldiers of the garrison of Leghorn have been taken ill, the hospitals being crowded with patients. The physicians believe that influenza has re-appeared.

The Duchess of Albany has gained certificates from the St. John Ambulance Association, for the two preliminary examinations for nursing and first aid to the injured.

Professor Liebreich, of Berlin, asserts that he has discovered a cure for tuberculosis more effective than Dr. Koch's, and says that the method of administration is similar.

An entertainment was given in Brompton Hospital on February 17th by Miss Mary Howel and other ladies. The programme was well carried out and reflected great credit on all concerned.

The Leeds Work-people's Hospital Fund amounted in 1890 to £5,261 14s. 1d. This sum, less £313 3s. 6d. for working expenses, has been distributed among the medical charities of Leeds.

Seventy-five persons were treated in Lytham Cottage Hospital and Convalescent Home during 1890. The expenditure amounted to £327 12s. 4d., and the total receipts to £380 2s. 4d., leaving a balance of £52 10s.

The Infectious Diseases Hospital in the Borough of St. Helen's has been declared free. The charge of 1s. a-day will no longer be charged. Patients who desire to pay will be charged the full cost of their maintenance, viz., 3s. a-day.

The twenty-third annual dinner in aid of the funds of the French Hospital and Dispensary was held on February 7th at the Hotel Métropole, M. Waddington, the French Ambassador, presiding. Subscriptions amounting to £2,500 were announced.

The Gresham Committee of the Mercers' Company have granted permission to the Hospitals' Association to establish an ambulance station within the portico of the Royal Exchange. This is the fiftieth station which the Association has established in the metropolis.

The income of the East Grinstead Cottage Hospital for 1890 was only £408 5s. 8d., but out of this sum the Committee find themselves in a position to add £100 to the capital account, and have £40 in hand for current expenses 47 patients were under treatment during the year.

The lady doctors in Edinburgh have achieved a victory of some moment, for they have succeeded in gaining admission to the Royal Infirmary for the purposes of clinical instruction. Though guarded round with numerous restrictions, the concession is an important one.

The complete returns now issued for 1890 show that the number of paupers in England and Wales was highest in the first week in March, and lowest in the second week in October. The number at the end of the year was actually smaller than at the same period in any year since 1887.

The annual report for 1890 of the Bradford Infirmary and Dispensary states that 1,982 in-patients, 2,475 home-patients, 6,303 out-patients, and 2,336 casualties have been dealt with. The total expenditure for the year was £8,076 11s. 1d., a reduction of £923 on that of 1889. The receipts amounted to £12,924 16s. 11d., of which sum £1,718 19s. 11d., were legacies for investment.

The forty-second annual report of the Portsmouth Hospital, states that during 1890, 937 in-patients and 6,978 out-patients received treatment at the hospital. The expenditure for the year was £6,375 2s. 5d. while the income was £6,056 1s. 1d., leaving an adverse balance of £320 1s. 4d. £2,000 which was raised by a bazaar in October, has been added to the investments of the hospital.

The annual meeting of the subscribers of the Nottingham Hospital for Women was held on February 11, Mr. F. Smith presiding. The report stated that 75 in-patients and 631 out-patients attended during the year. The balance-sheet showed the income to have been £685 19s. 6d., and the expenditure £742 6s. 6d. The receipts on the building fund amounted to £2,278 8s. 5d. and the expenditure to £2,084 6s. The report and balance-sheet were passed. The meeting then ended.

### THE STUDENT OF MEDICINE.

[All communications for this Supplement should be addressed to THE EDITOR of THE HOSPITAL, at 140, Strand, with the words, "Students' Column," written in the left hand top corner of the envelope.]

### LEADING ATHLETES.

We publish this week the portrait and short biographical sketch of Mr. Ernest Wool Lewis, the well-known lawn tennis player. He was born at Hammersmith in the year 1867, and educated at the Godolphin School, Hammersmith. After leaving school he entered St. George's Hospital as a student of medicine in 1886. When a school-boy he was fairly good at open court rackets and bat-fives, and was also a good runner, but never competed in any races. Since his school days lawn tennis is the only athletic game that he has gone in for. His first success was at Eastbourne, in 1882, when he won the open championship, receiving half 15 and a bisque from the late Donald Stewart (scratch). Since this time Mr. Lewis has won a large number of championships and challenge cups, some of the more important being as follows: The double championship of Scotland with R. M. Watson in 1885; the South of England Championship in 1886-7 and 8; the Bournemouth Challenge Cup in 1883-4-5-6-7-8, and 9; the London Championship in 1886 7 and 8; the Torquay Challenge Trophy in 1887-8 (1889 there was no competition) and 1890; the Middlesex Championship in 1887-8-9 and 90; the Covered Courts Championship in 1887-8-9 and 90; the Exmouth Challenge Plate in 1888-9 and 90; the Staffordshire Championship in 1888; the South Saxons Challenge Cup in 1889. He also won the Irish Mixed Doubles Championship in 1888, his partner being Miss M. Bramwell; the Irish Gentlemen's Doubles Championship with G. W. Hilliard in 1889. In 1890 he also won the Irish Gentlemen's Open Single Championship, defeating consecutively F. O. Stocker, D. G. Chaytor, J. Pim, and W. J. Hamilton. In 1889 he was the winner of the All Comers and Challenge for the Welsh Championship. In both the years 1886 and 1888 Mr. Lewis was runner-up for the English Championship. He is 5 ft. 9½ in. high, and 11 stone in weight.

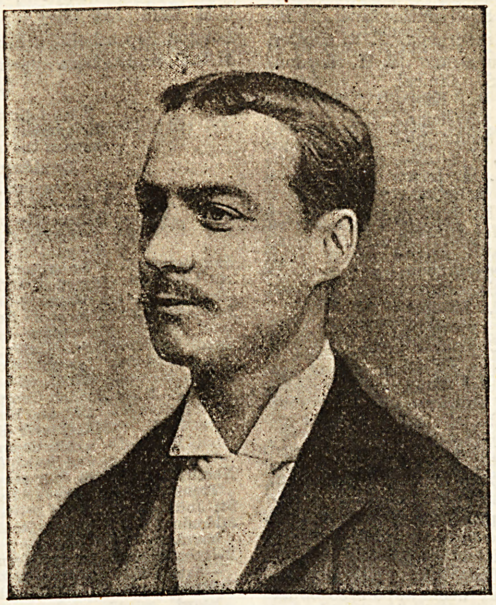

*From a photograph by Walery.*

MR. E. W. LEWIS (St. George's).

THE STUDENT OF MEDICINE—(*continued*).

### APPOINTMENTS.

At St. Thomas's Hospital, F. W. Belville, L.R.C.P., M.R.C.S., has been appointed clinical assistant for diseases of the skin. W. R. Carter, B.A. Cantab., M.R.C.S., L.R.C.P., resident accoucheur. T. A. M. Forde, L.R.C.P., M.R.C.S., clinical assistant for diseases of the skin. W. S. Griffith, M.A., M.B., B.C.Cantab., L.R.C.P., M.R.C.S., house surgeon. J. R. Harper, M.R.C.S., L.R.C.P., assistant house surgeon. T. H. Haydon, M.B., B.C.Camb., L.R.C.P., M.R.C.S., clinical assistant for diseases of the ear. A. King, L.R.C.P., M.R.C.S., resident house physician. H. D. Levick, M.R.C.S., L.R.C.P., senior obstretric clerk. C. P. Lovell, M.A.Oxon., M.R.C.S., L.S.A., non-resident house physician. H. Low, M.A., M.B., B.C.Camb., M.R.C.S., L.R.C.P., non-resident house physician. W. H. Millar, L.R.C.P., M.R.C.S., clinical assistant for diseases of the throat. L. A. J. Roulliard, M.B., B.C.,Camb. L.R.C.P., M.R.C.S., house surgeon. A. W. F. Sayres, L.R.C.P., M.R.C.S., clinical assistant for diseases of the ear. D. F. Shearer, B.A., B.M., B.Ch.Oxon., house surgeon. W. G. G. Stokes, B.A.. M.B., B.C. Cantab., L.R.C.P., M.R.C.S., house surgeon. S. G. Toller, L.R.C.P., M.R.C.S., house surgeon. W. F. Umney, L.R.C.P., M.R.C.S., resident house physician, and C. H. Usher, B.A., M.B., B.C.Camb., clinical assistant for diseases of the throat. At the Children's Hospital, Great Ormond Street, Horace Collier, F.R.C.S., L.R.C.P., has been appointed house physician. At the St. Pancras Infirmary, Dartmouth Park Hill, J. E. Creswell, L.R.C.P., M.R.C.S., has been appointed assistant medical superintendent. At the London FeverHospital, William Hunter, M.D., M.R.C.P., as been appointed assistant physician. At the City of London Hospital for Diseases of the Chest, Victoria Park, W. W. Simmonds, M.B.,Cantab., has been appointed house physician. At the Ingham Infirmary and South Shields and Westoe Dispensary. Collin Gordon, M.B., C.M.Edin., has been appointed senior house surgeon. At the Scarborough Hospital and Dispensary, Victor Hodgson, M.R.C.S., L.R.C.P., has been appointed house surgeon. At the Macclesfield General Infirmary, Oswald Rees, M.B., C.M.Glas., has been appointed junior house surgeon. At the Hospital for Consumption and Diseases of the Chest, Brompton, W. Tweeddale Thompson, L.A., M.B., C.M. Edin., has been appointed house physician. At the Central London Op hthalmic Hospital, J. Cecil Tosswill, L.R.C.P., M.R.C. S., has been appointed house surgeon.

### VACANCIES.

The following vacancies are announced this week, the amount of salary in each case being given after the name of the institution:

**Resident Medical Officers.**

City Asylum, Birmingham, Clinical assistant—Board, &c.

General Hospital, Birmingham, Resident Medical Officer—Salary £130.

Hospital for Consumption and Diseases of the Chest, Brompton, House Physicians—Board, &c.

Liverpool Infirmary for Children, Myrtle Street, House Surgeon—Salary £85, and board, &c.

Liverpool Royal Southern Hospital, Junior House Surgeon—Salary 60 guineas, and board, &c.

Leeds Union, Assistant Medical Officer and Dispenser for the Work-house, Schools, and Infirmary—Salary £100, and board, &c.

London Temperance Hospital, House Surgeon—Salary £50, and board, &c.

Manchester Royal Infirmary, Resident Medical Officer to the Convalescent Hospital at Cheadle—Salary £150, and board, &c.

North Staffordshire Infirmary and Eye Hospital, Hartshill, Stoke-upon-Trent, Assistant House Surgeon—Board, &c.

St. John's Hospital for Diseases of the Skin, House Surgeon—Board.

Addenbrooke's Hospital, Cambridge, House Physician—Salary £65, and board.

Brixton, Streatham, and Herne Hill Dispensary, Resident House Surgeon—Salary £150, and board, &c.

Cheltenham General Hospital, Junior House Surgeon—Salary £40, and board.

Chichester Infirmary, House Surgeon—Salary £80, and board, &c.

County Donegal Infirmary, House Surgeon and Registrar—Salary £60, and board, &c.

THE STUDENT OF MEDICINE—(*continued*).

National Dental Hospital, Great Portland Street, W., House Surgeon—Board, &c.
Royal Victoria Hospital, Bournemouth, House Surgeon and Secretary—Salary £80, and board, &c.
West Bromwich District Hospital, Assistant House Surgeon—Board, &c.

**Non-Resident Medical Officers.**

Brown Animal Sanatory Institution, Professor Superintendent—Salary £250.

St. George's and St. James's Dispensary, Surgeon.

Grosvenor Hospital for Women and Children, Westminster, Surgeon.

Owens College, Manchester, Demonstrator in Pathology—Salary £140.

Royal Academy of Arts, Professor of Anatomy.

St. Thomas's Hospital, Surgeon.

St. Thomas's Hospital, Assistant Physician.

Great Northern Central Hospital, Holloway Road, Surgeon to the Out-patients.

### PASS LISTS.

### London, February 11th.

PRELIMINARY SCIENTIFIC (M.B.) EXAMINATIONS.

ENTIRE EXAMINATION.

*First Division:* William Dyche, B.A., Westminster Training and Yorkshire Colleges; Thomas H. Green, Guy's Hospital; Richard Kay, Owens College; Joseph A. Mawson, Yorkshire College; Harold B. Shaw, Yorkshire College; Arthur H. Spicer, The Leys, Cambridge, and University College. *Second Division:* Malcolm Cameron, London Hospital; Claude L. Chevalier, St. Thomas's Hospital and Birkbeck Institution; Horace J. Harris, B.A., University College; Thomas C. Last, St. Mary's Hospital; Cuthbert H. J. Lockyer, St. Thomas's Hospital and Birkbeck Institution; William J. Oakes, B.A.; Percy A. Palmer, St. Thomas's and St. Bartholomew's Hospitals; George H. Wheeler, B.A.

CHEMISTRY AND EXPERIMENTAL PHYSICS.—Algernon C. Bean*. University College; Sydney H. Belfrage*, University College; William M. Bergin*, the Clifton Laboratory; Victor J. Blake*, University College; Herbert Cardin, Dulwich College; Felix B. Carter*, University College; Walter E. Cross*, Guy's Hospital; Edward C. Davenport, Mason College; Alfred Dimsey*, University College; Stewart R. Douglas*, St. Bartholomew's Hospital; Edward G. Drury*, University College; B. Dyball, St. Thomas's Hospital and Birkbeck Institution; Arthur Earnshaw*, King's College, Guy's Hospital, and Birkbeck Institution; William D. Frazer*, University College, Cardiff; Frederick H. Gervis, St. Paul's School and St. Thomas's Hospital; Henry N. Goode, St. Thomas's Hospital and Birkbeck Institution; Charles Hanks; Charles J. Harnett*, Guy's Hospital; Thomas Hood*, St. Bartholomew's Hospital and City of London College; David Horwitch*. Mason and Queen's Colleges, Birmingham; Edmund E. Lloyd, Leonard.F Marks*, Joseph T. Marsden, Owens College: Sydney H. Mason*, Mason College; Joseph C. Muir*, the Leys School, Cambridge; William P. Nicol*, King Edward's High School, Birmingham; James H. F. Nunn*, St. Bartholomew's Hospital and Birkbeck Institution: Arthur J. Pedley*, Mason College and King Edward's High School, Birmingham; Norman H. Pike*, Dulwich College; David Rice*, Guy's Hospital; Edward L. M. Rushby*, King's College; Joseph Shardlow*, Firth College, Sheffield; Frank A. Smith*, St. Bartholomew's Hospital; Lewis A. Smith*, London Hospital; and James C. Spillane*, London Hospital; Leonard K. Thomas*, King Edward's High School, Birmingham; Charles R. Watson, St. George's Hospital; Henry C. Wett, University College, Liverpool; Morris Wilks*, University College; Harry M. Wise*, Guy's Hospital.

BIOLOGY.—William B. Bennett*, University College, Liverpool; Lewis W. Burrow*, private study; Joseph E. G. Calverley*, Dulwich College and St. Bartholomew's Hospital; Cecil F. Gordon*, Dulwich College and St. Bartholomew's Hospital; J. Alban K. Griffiths*, University and Epsom Colleges; Thomas A. Hawkesworth, private study; Sindia Emilz Hickson, University Colleges, London and Bristol; Albert Hilton*, Owens College; Gwilym P. James, University College, Aberyst.; Arthur D. Ketchen, University College; William M. M'Donald, St. Bartholomew's Hospital; F. Archibald H. Michod, St. Mary's Hospital; Charles H. Murray, Guy's Hospital; Herbert A. Scott*, Owens College; Thomas B. Sellors*. Middlesex Hospital; Arthur R. H. Skey*, St. Bartholomew's Hospital; John C. Smellie*, St. Mary's Hospital; Daniel L. Smith, University College, Aberyst., and Guy's Hospital; Francis J. Smith, Owens College; Graham U. Smith*, King's College; Percy M. Smith*, Epsom College and St. Mary's Hospital; Harry J. Spon*, Guy's Hospital; John R. Steinhauser*, Guy's Hospital; Cecil W. Stickland*, St. Paul's School; Thomas M. Thomas*, University College, Aberyst. and Guy's Hospital; Edward O. Thurston*, private study; Sydney G. Tippett*, St. Mary's Hospital; Thomas H. Wells*, private study and tuition; Stanley Whicher, University College, Aberyst., and Guy's Hospital.

TWO SUBJECTS OF THE EXAMINATION (under former regulations) * †.—Colin C. Clarkson (C., B.), St. Thomas's Hospital and University College; A. William R. Cochrane (P., B.). St. Bartholomew's Hospital; Adèle I. De Steiger (C., P.), University and Bedford Colleges, London; William Henry Gray (P., B.); Thomas Lambe (C., P.), University College; Eldon Pratt (C., P.), St. Bartholomew's Hospital and Birkbeck Institute.

ONE SUBJECT OF THE EXAMINATION (under former regulations) * †.—William Edward N. Dunn (B.), St. Bartholomew's Hospital; Henry W. Eldred (B.), St. Thomas's Hospital; Ernest J. Hynes (B.), London Hospital; Harry F. Turner (C.), Guy's Hospital.

* These candidates have now completed the examination.
† The subjects taken up by these candidates are indicated by initials after the names: C., chemistry; P., physics; B., biology.

### THE STUDENT OF MEDICINE—(*continued*).

### Indian Medical Service.

The following is a list of surgeons on probation in her Majesty's Indian Medical Service who were successful at both the London and Netley Examinations:—

| | Marks. | | Marks. |
|---|---|---|---|
| *Crawford, J. M.... | 6,100 | ‡O'Kinealy, F. ... | 5,580 |
| †Wolfe, J. W. ... | 5,735 | Cassidy, C. C. ... | 5,540 |
| Singh, B. J. ... | 5,655 | Murray, F. E. ... | 5,520 |
| Whitcombe, E. G. R. ... | 5,655 | Buist-Sparks, A. W. T. | 5,500 |
| Barber, H. R. C. ... | 5,645 | Younger, H. J. ... | 5,490 |
| James, C. H. ... | 5,638 | Basu, B. D. ... | 5,160 |

* Gained the Prize in Clinical Medicine presented by Surgeon-General W. C. Maclean, C.B., with the De Chaumont Prize in Hygiene, and the Montefiore second Prize.
† Gained the Martin Memorial Gold Medal.
‡ Gained the Prize in Pathology presented by Sir William Aitken, F.R.S.

### British Medical Service.

The following is a list of surgeons on probation of the Medical Staff of the British Army who were successful at both the London and Netley examinations:—

| | Marks. | | Marks. |
|---|---|---|---|
| §Beach, T. B. ... | 5,927 | Hardy, F W. ... | 5,102 |
| Powell, E. E. ... | 5,460 | Clapham, J. T. ... | 5,035 |
| Healey, C. W. R. ... | 5,325 | Graham, W. Ap. S. J.... | 4,995 |
| Jennings, J. W. ... | 5,265 | Shanahan, D. D. ... | 4,975 |
| Williams, E. McK. ... | 5,197 | Whitestone, C. W. H. ... | 4,940 |
| Dowse, H. E. ... | 5,183 | Pearse, A.... ... | 4,880 |
| Connor, J. C. ... | 5,118 | D'Alton, C. ... | 4,643 |
| Carter, J. E. ... | 5,106 | Mason, H. D. ... | 4,640 |

§ Gained the Herbert Prize of £20, with the Montefiore Medal and Prize of 20 guineas.

### EVENTS FOR THE MONTH OF MARCH.

#### Students' Medical Societies.

St. Bartholomew's Hospital Abernethian Society.—March 5th, Mr. R. A. Bickersteth, M.B., on the Treatment of Wounds. March 12th, Mr. J. H. Drysdale, on Diphtheritic Paralysis.

St. George's Hospital Hunterian Society.—March 13th, Clinical Evening. March 27th, Mr. Frost on Lantern Demonstrations on the Fundus of the Eye.

Guy's Hospital Pupils' Physical Society.—March 7th, Clinical Evening. March 21st, Treasurer's Prize Essay.

St. Mary's Hospital Medical Society.—March 4th, Ophthalmic Demonstrations, Mr. H. Juler and Mr. J. Griffith. March 18th, Clinical evening.

Middlesex Hospital Medical Society.—March 12th, Special Sense Organs.

St. Thomas's Hospital Medical and Physical Society.—March 5th, Mr. E. Millais, on Distemper in the Dog and Experimental Work thereon.

University Hospital Medical Society.—March 11th, Paper by G. Pernet, "Some Flashes of Mediæval Knowledge." Demonstrations: Pathological—Diseases of Women; Histological—Changes in Female Organs; chairman, Professor H. C. Bastian, M.A., M.D., F.R.S. March 25th, Clinical and Pathological Evening. Demonstrations: Pathological—Inflammation; Histological—Blood. Chairman, the President

Westminster Hospital Guthrie Society.—March 12th, Meeting at eight p.m.

#### Athletics.

Football Matches.

St. Bartholomew's Hospital.—March 7th, England v. Scotland, London. Second team: March 21st, v. Marlborough Nomads, at Surbiton.

Guy's Hospital.—Rugby: first fifteen, March 7th, England v. Scotland, London; and v. Blackheath, at Rectory Field. Second fifteen: March 7th, England v. Scotland, London. Association: first eleven, March 11th, v. City Ramblers, at Raynes Park; 14th, v. Chiswick Park, at Chiswick Park.

King's College.—Association: March 21st, v. St. John's Hall, at Highbury. Second eleven: March 4th, v. University College second eleven, at Wormwood Scrubbs.

London Hospital.—Association: March 7th, v. Beckenham, at Beckenham.

St. Thomas's Hospital.—March 4th, v. Casuals, at Upper Tooting; 7th, v. St. Margaret's, at St. Margaret's; 14th, v. Westminster Club, at Wandsworth; 21st, v. Chiswick Park, at home.

St. Thomas's Hospital.—March 7th, England v. Scotland, in London; 14th, v. R.I.E.C., at Cooper's Hill. Second fifteen: March 14th, v. Clapham Rovers second fifteen, at Wandsworth.

University College and Hospital.—First team: March 14th v. Surbiton F. C., at Surbiton; 21st, v. Royal School of Mines, at Neasden.

Westminster Hospital.—March 7th, v. Christ's Hospital, at Balham. Cup ties.

Owens College.—First team: March 4th, v. Birkenhead Park, away. A team: March 7th, v. Ashford House F. C., at home; 21st, v. Horwich, away; 28th, v. Broughton Rangers, away. B team: March 7th v. Broughton third, away; 14th, v. Liverpool Old Boys (B), at home; 21st, v. Matfield (A), at home; 28th, v. Blackley, at home.

#### United Hospitals Hare and Hounds.

March 7th, Challenge Cup contest, at Raynes Park; 14th, ordinary run at Raynes Park.

